# The After-Effects of Lumbar Puncture

**Published:** 1919-08-16

**Authors:** 


### THE AFTER-EFFECTS OF LUMBAR PUNCTURE.

Although the technique of this small operation is by now well known, there are a few puzzling phenomena which may occur, and one of the least understood is the so-called *puncture headache*. A curious fact may be noted. The throbbing pain, most severe in the frontal and occipital regions, is only present when the patient is sitting up, and disappears completely when he lies down. Hunt points out that the pain comes on quickly when the man sits up, appearing in about twenty seconds and subsiding again in about the same time when he lies back on his pillow.

A number of causal factors are suggested—rapidity of withdrawal, the position during puncture, the degree of intraspinal pressure, the disease present, the age and general condition of the patient—but no satisfactory decision has been reached.

R. G. McRobert in America offers an ingenious explanation. The needle must in any case puncture two membranes—the dura and the arachnoid. The former is just within the vertebral canal, and forms a tough fibrous membrane, piercing of which leaves a clean-edged round hole. The needle passing through the arachnoid enters a contracting membrane, which readily closes after the steel is withdrawn, and then fits tightly up against any hole persisting in the dura, since the punctures being very small they are most unlikely to coincide. The author suggests that under certain circumstances the soft arachnoid membrane may cling round the departing needle and be drawn through the hole in the dura, there persisting as a tiny funnel or wick through which for some days cerebrospinal fluid may leak from the canal. For a short time all the fluid secreted by the choroidal mechanism may be drained away and absorbed, the amount originally obtained in the operator's tube being no indication of the quantity actually lost. The epidural space of the spinal canal is very large and contains only loose connective tissue, venous and lymph plexuses, which can dispose of a large amount of fluid without any external signs.

Reduction of amount of available cerebrospinal fluid and a diminished "protective water jacket" of the brain, partially compensated when the patient lies down, would possibly account for the peculiar nature of the headache. Also one may see a strong indication for the use of perfectly sharp and polished lumbar-puncture needles if the above be indeed the mechanism of hidden leakage.

